# Imaging of urgencies and emergencies in the lung cancer patient

**DOI:** 10.1007/s13244-018-0605-6

**Published:** 2018-04-11

**Authors:** Bruno De Potter, Jef Huyskens, Birgitta Hiddinga, Maarten Spinhoven, Annelies Janssens, Jan P. van Meerbeeck, Paul M. Parizel, Annemie Snoeckx

**Affiliations:** 1 Department of Radiology, University Hospital Antwerp and University of Antwerp, Wilrijkstraat 10, 2650 Edegem, Belgium; 2 Department of Thoracic Oncology, University Hospital Antwerp and University of Antwerp, Wilrijkstraat 10, 2650 Edegem, Belgium; 3 Department of Thoracic Oncology, University Medical Centre Groningen, Hanzeplein 1, 9713 GZ Groningen, Netherlands

**Keywords:** Lung cancer, Emergencies, Radiography, Computed tomography, Magnetic resonance imaging

## Abstract

**Abstract:**

Lung cancer patients often experience potentially life-threatening medical urgencies and emergencies, which may be a direct or indirect result of the underlying malignancy. This pictorial review addresses the most common thoracic, neurological and musculoskeletal medical emergencies in lung cancer patients, including superior vena cava syndrome, pulmonary embolism, spontaneous pneumothorax, cardiac tamponade, massive haemoptysis, central airway obstruction, oesophagorespiratory fistula, malignant spinal cord compression, carcinomatous meningitis, cerebral herniation and pathological fracture. Emphasis is placed on imaging findings, the role of different imaging techniques and a brief discussion of epidemiology, pathophysiology and therapeutic options. Since early diagnosis is important for adequate patient management and prognosis, radiologists have a crucial role in recognising and communicating these urgencies and emergencies.

**Teaching points:**

*• Multiplanar multidetector computed tomography is the imaging examination of choice for thoracic urgencies and emergencies.*

*• Magnetic resonance imaging is the imaging modality of choice for investigating central nervous system emergencies.*

*• Urgencies and emergencies can be the initial manifestation of lung cancer.*

*• Radiologists have a crucial role in recognising and in communicating these urgencies/emergencies.*

## Introduction

Lung cancer is very common, accounting for 17% and 9% of all cancers in men and women, respectively [1]. Furthermore, lung cancer is the biggest cancer killer, representing 19% of all cancer-related deaths. The disease course in lung cancer is generally characterised by high morbidity and complications, some of which are acute and potentially life-threatening. An oncological emergency can be defined as an acute, potentially life-threatening condition in a cancer patient, either as a direct or indirect effect of the underlying malignancy or secondary to its treatment, requiring rapid intervention to avoid death or severe morbidity. Urgencies and emergencies differ in the severity of the consequences of a delay in treatment. Oncological emergencies can occur at any time during the course of a malignancy and can be the initial manifestation in some patients [2]. The causes of oncological urgencies and emergencies are myriad. While metabolic, infectious and haematological emergencies are primarily diagnosed by the clinical presentation and laboratory findings, thoracic, neurological and musculoskeletal emergencies often require imaging studies. The aim of this pictorial review is to identify and discuss these urgencies and emergencies in which radiologists play an important role and can have a significant impact on patient management and prognosis. The contribution of imaging in urgencies and emergencies goes beyond making the initial diagnosis, as imaging can also play a role in the planning of treatment and follow-up after treatment.

## Thoracic emergencies

### Superior vena cava syndrome

**Fig. 1:**
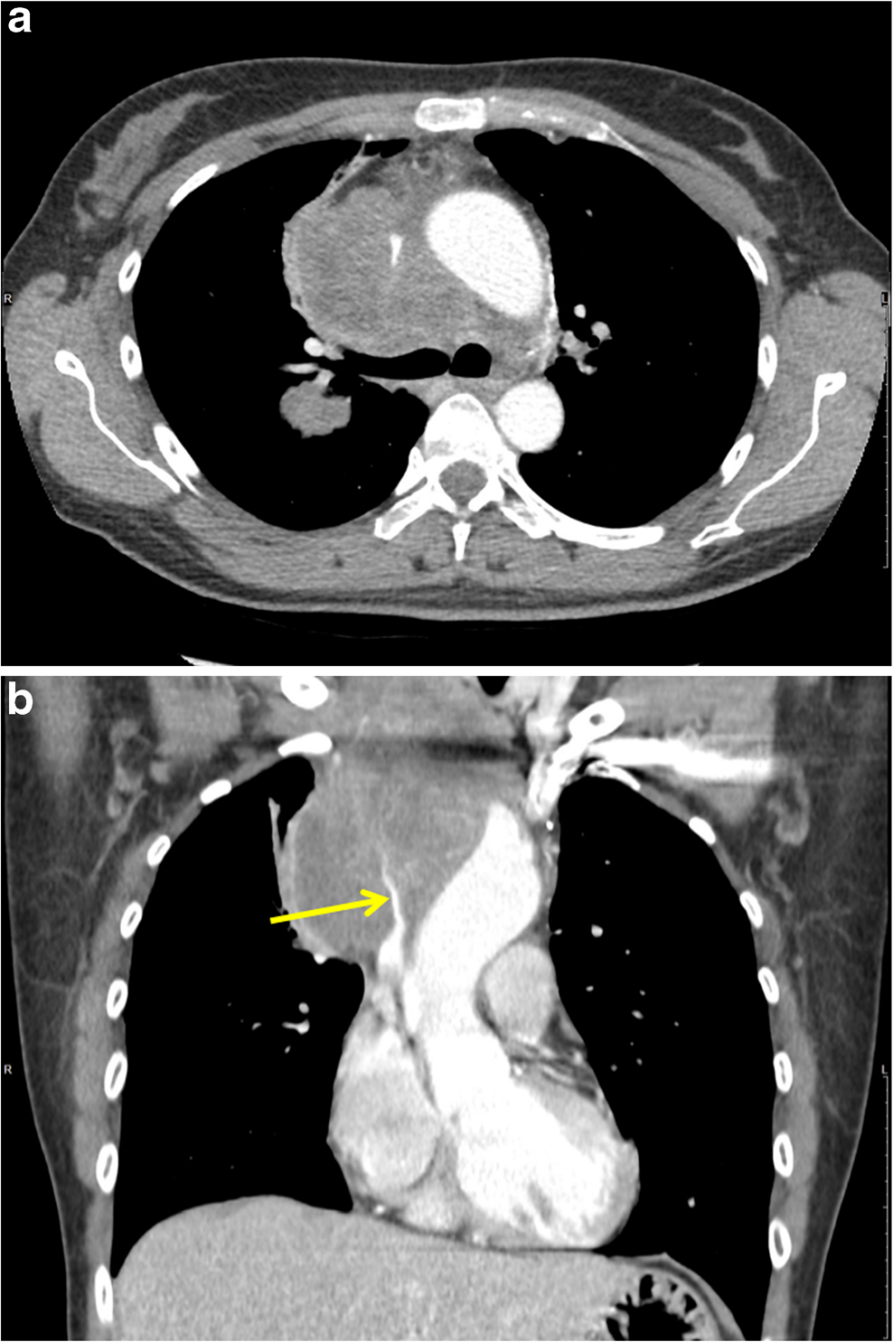
Superior vena cava syndrome (SVCS) as the initial presentation of a stage IIIB small cell lung cancer in a 48-year-old woman. **a** Contrast-enhanced axial CT image in mediastinal window setting depicts a large mass located in the visceral mediastinal compartment with encasement of mediastinum and in particular the SVC. **b** CT image reconstruction in the coronal plane shows the large soft tissue mass and better depicts the prominent narrowing of the SVC (*yellow arrow*)

Superior vena cava syndrome (SVCS) consists of various symptoms arising from dysfunction of the superior vena cava. While the cause of SVCS can be infectious, inflammatory, thromboembolic or malignant, malignancies account for up to 90% of causes. In the setting of malignancy, obstruction can be caused by either invasion or external compression of the superior vena cava (SVC) by a pathological process or by (coexistent) thrombosis of blood within the SVC. SVCS affects up to 10% of small cell lung cancer (SCLC) patients and up to 2–4% of all lung cancer patients [3]. SVCS is determined by increased venous pressure in the upper body from the SVC obstruction and manifests with easily discernible symptoms on clinical examination, including oedema of the head, neck, eyelids, upper torso, arms and distinctly dilated veins. Laryngeal and pharyngeal oedema may cause narrowing of the respiratory tract [4]. Chest radiograph may show a bulky mass, whereas computed tomography (CT) with intravenous contrast is the imaging modality of choice for more detailed visualisation of the SVC and to depict the relationship of the tumour with the SVC (Fig. 1). For optimal evaluation of the SVC, CT of the chest is best perfomed 60 s after peripheral intravenous injection of 120 mL iodinated contrast at a rate of 3 mL/s [5]. However, SVCS is frequently diagnosed on routine contrast-enhanced chest CT. In case of thrombosis, CT shows a filling defect in the superior vena cava, often caused by direct invasion by a malignancy arising from the lungs or mediastinum. Streak artefacts due to non-enhanced blood from contralateral veins should not be mistaken for a thrombus. Secondary signs such as a collateral vascular network may also point to the diagnosis of an occluded or compressed SVC. On CT, these collateral veins appear as densely opacified tortuous vascular channels. The most commonly visible venous collateral network is the azygos and hemiazygos system, which is an important ancillary CT finding of SVCS. Other important thoracic venous collaterals are the vertebral and subscapular plexuses, the mediastinal, oesophageal, and diaphragmatic venous plexuses, and the lateral thoracic and superficial thoracoabcominal venous plexuses [6]. Coronal reformatted images are often helpful to delineate the extent of the SVC thrombus or compression. Patients with lung cancer presenting with SVC, who need emergency treatment, should receive urgent chemoradiotherapy with or without endovascular stenting for rapid symptom relief as well as to optimise overall outcome and prevent recurrence of SVCS [4].

### Massive pulmonary embolism (PE)

**Fig. 2:**
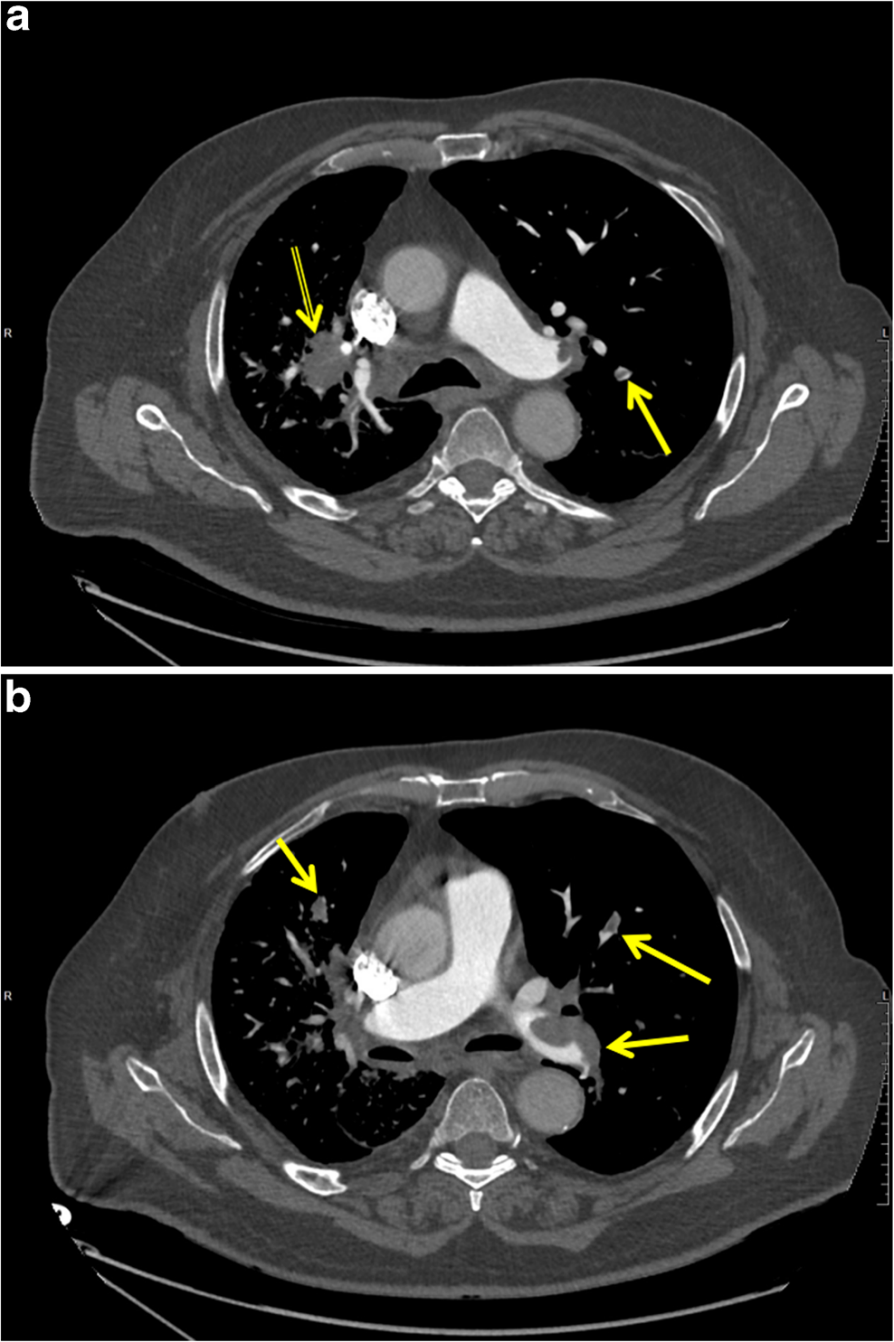
Pulmonary embolism in a 73-year-old man with known stage IV non-small cell lung cancer. Axial reconstructions of a CT pulmonary angiography study at the carinal (**a**) and infracarinal (**b**) level showing a hilar mass (*double yellow arrow*) in the right upper lobe (**a**) and large emboli involving the left main pulmonary arteries as well as bilateral subsegmental emboli (**a**, **b**) (*yellow arrows*)

Compared to the general population, cancer patients carry a higher risk for development of venous thromboembolism (VTE), including PE and deep venous thrombosis (DVT). It is estimated that cancer is responsible for 20% of all cases of VTE and lung cancer is amongst the malignancies with the highest incidence rates [7]. Risk factors for VTE are advanced disease, chemotherapy treatment and treatment with anti-angiogenic agents [7]. Prevalence of asymptomatic PE in lung cancer outpatients has been estimated to be 14.9% [8]; consequently, diagnosis of incidental PE when contrast-enhanced CT is performed for other indications is relatively common. Clinical presentation of patients with PE is similar regardless of the cause of the pulmonary emboli, with symptoms of dyspnoea, chest pain and signs of right heart failure. The severity of the symptoms is related to the extension of embolism, the size of the clots and underlying heart or lung disease. If properly diagnosed and treated, there is no significant difference in survival rate between lung cancer patients with and without PE, and most deaths are attributable to disease progression [9]. However, because symptoms of PE such as dyspnoea and chest pain are non-specific and particularly common in lung cancer patients, the presence of PE is easily overlooked clinically, and radiologists play an important role in picking up incidental PE. CT pulmonary angiography (CTPA) is the imaging modality of choice and will show filling defects in the pulmonary vasculature (Fig. 2); when observed in the axial plane, this has been described as the “polo mint” sign. When pulmonary emboli are present, attention should be paid to the heart. Imaging findings that suggest right ventricular failure include right ventricular dilatation with or without contrast material reflux into the hepatic veins and/or deviation of the interventricular septum toward the left ventricle [10]. A right ventricle/left ventricle short axis ratio greater than 1 on reconstructed four-chamber views is indicative of right heart failure and a bad prognostic sign [11]. Peripheral wedge-shaped areas in the lung parenchyma may indicate lung infarction and should alert the radiologist to pulmonary embolism as a possible underlying cause.

Haemodynamic and respiratory support is the initial treatment of acute massive PE, followed by anticoagulation or, in the case of massive acute PE, fibrinolysis [12]. Treatment for patients with incidental, asymptomatic PE remains the same as for patients with symptomatic PE according to guidelines published by the American College of Chest Physicians [13].

### Spontaneous pneumothorax

**Fig. 3:**
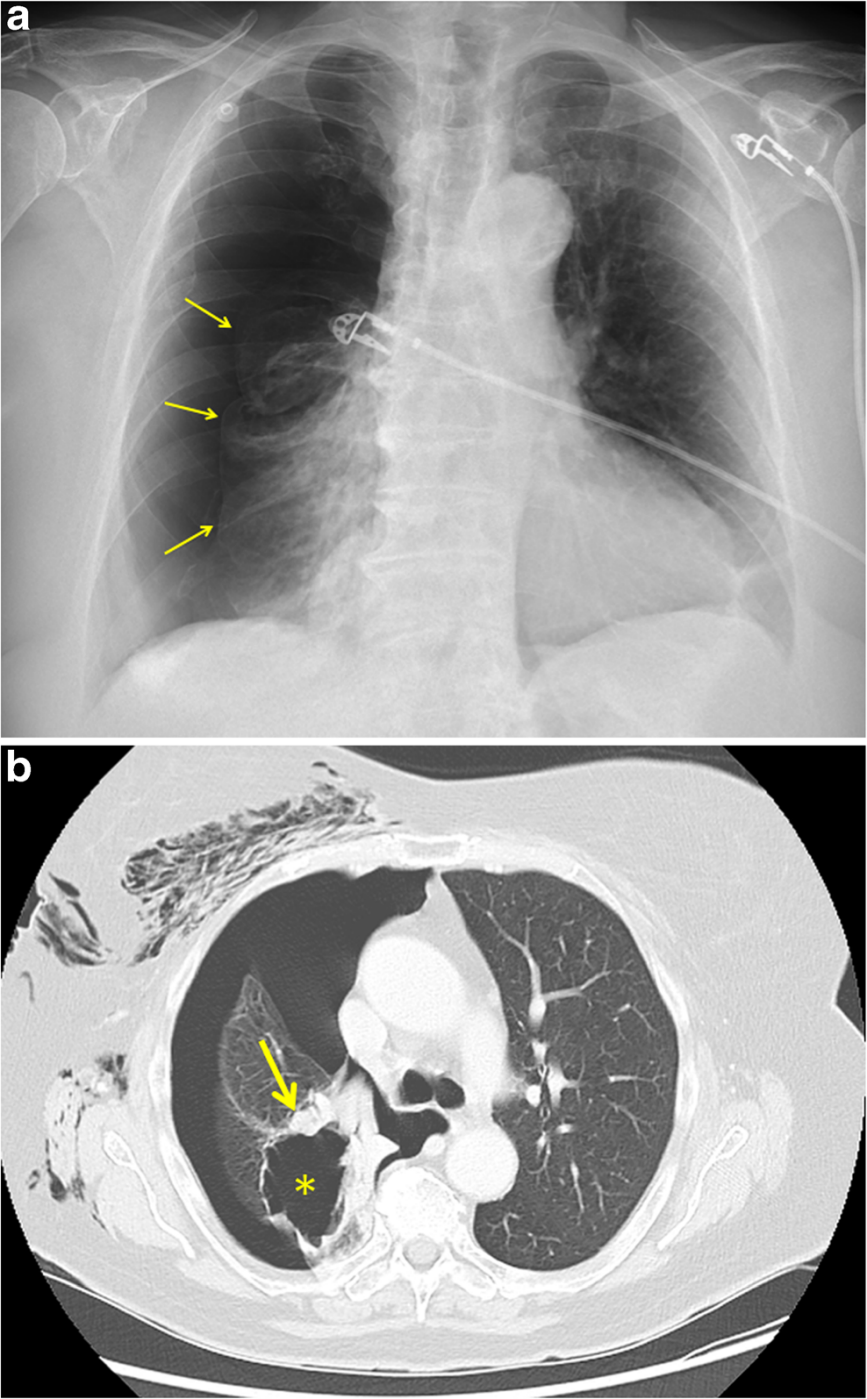
Spontaneous pneumothorax as the first sign of a primary lung carcinoma in an 87-year-old woman who presented at the emergency department with thoracic pain and severe respiratory distress. **a** Supine anteroposterior chest radiograph clearly depicts a right-sided pneumothorax with complete collapse of the right lung. The visceral pleural edge is observed clearly as a very thin, sharp line (*yellow arrows*) with the absence of vascular marking beyond the pleural line. **b** Axial contrast-enhanced chest CT in lung window setting shows a large thin-walled cystic lesion (*asterisk*) with peripheral solid nodular component (*yellow arrow*) in the right upper lobe as the underlying cause of the pneumothorax. Also, note the extensive subcutaneous emphysema in the right chest wall. Histopathological diagnosis of adenocarcinoma (type “lung cancer associated with cystic airspaces”) was made after lobectomy

Spontaneous pneumothorax is a very rare complication of lung cancer with an estimated occurrence rate of 0.03-0.05% in primary lung cancer. Only 2% of all spontaneous pneumothoraces is coexistent with malignant lung diseases [14]. In approximately 75% of these cases, pneumothorax is the presenting feature of lung cancer [15]. The clinical presentation is variable, depending on the extent of the pneumothorax, ranging from asymptomatic to extreme dyspnoea with hypotension and tachycardia in case of tension pneumothorax, which can be life-threatening. In tension pneumothorax, a positive pressure on mediastinal and intrathoracic structures can result in a reduced cardiac output with typical features of hypoxaemia and haemodynamic compromise [16]. Erect chest radiograph is the imaging modality of choice for the initial diagnosis of a pneumothorax. CT is considered as the “gold standard” for the detection of a small (and anterior) pneumothorax and size estimation. The differentiation of a large from a small pneumothorax is made by identification of a rim of >2 cm between the lung margin and chest wall at the level of the hilum [16]. Moreover, CT can better identify the relationship with underlying lung pathology, such as lung cancer (Fig. 3). Particularly in older patients presenting with a spontaneous pneumothorax, CT images should be scrutinised for malignancy as a possible cause. A tension pneumothorax should be suspected if there are additional features such as a mediastinal shift to the contralateral side or depression of the ipsilateral hemidiaphragm.

Small, asymptomatic pneumothorax can be treated conservatively. Larger, symptomatic pneumothorax requires active intervention by needle aspiration or chest drain insertion [16].

### Cardiac tamponade

Pericardial effusion in oncology patients may develop by four mechanisms: direct extension or metastatic spread, chemotherapeutic toxicity, radiation toxicity, or as an opportunistic infection [17]. Primary lung cancer is the most common cause, accounting for over one-third of malignant pericardial effusions. Only a small percentage of patients with a malignant pericardial effusion develop cardiac tamponade, which is a medical emergency [18]. Cardiac tamponade results from an accumulation of pericardial fluid leading to an impaired ventricular filling with decreased cardiac output. This can occur with as little as 200 ml of pericardial fluid [17, 19]. Symptoms suggestive of cardiac tamponade are dyspnoea, non-specific chest pain and fatigue.

**Fig. 4:**
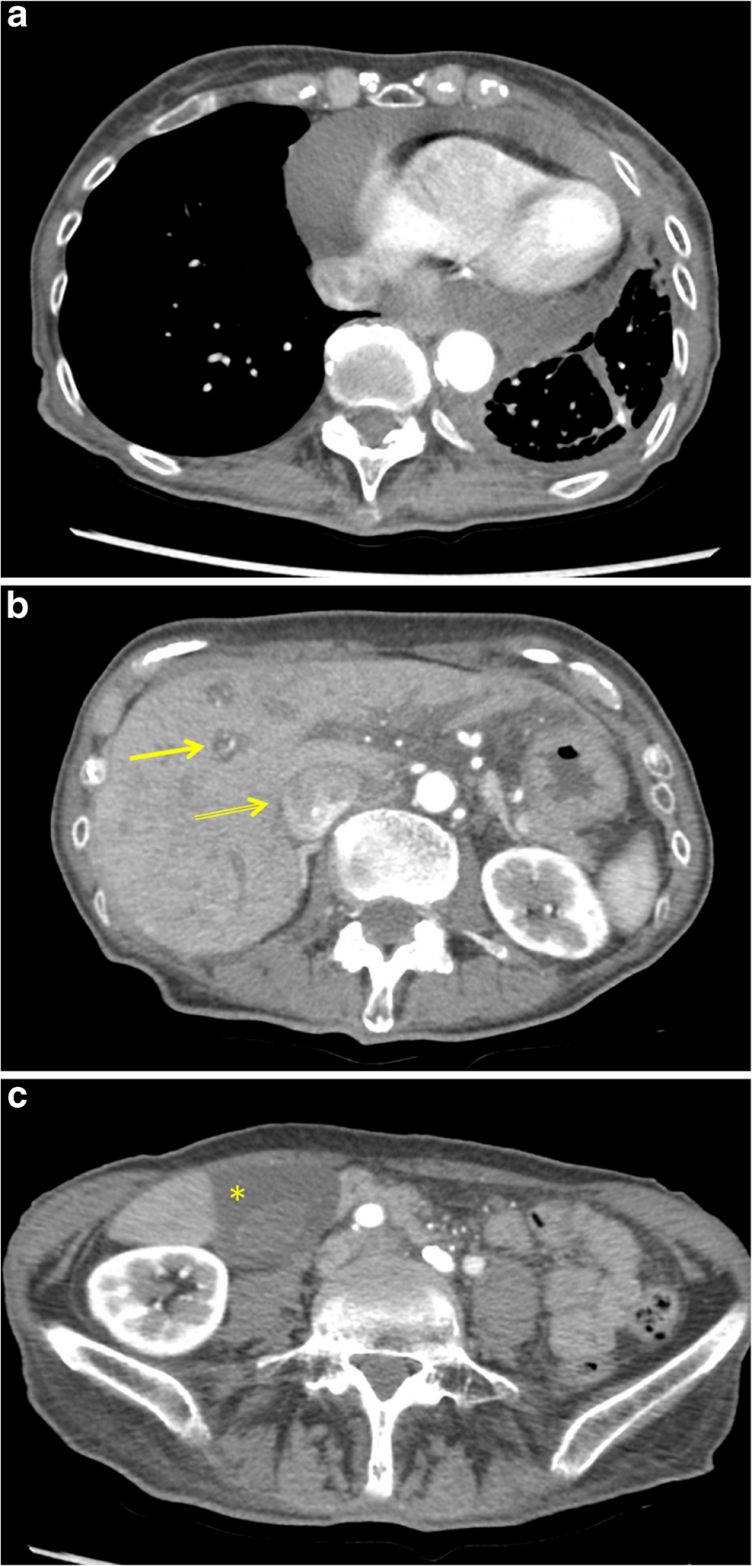
A 73-year-old woman with a known stage IV non-small cell lung cancer presented during follow-up with symptoms of chest pain, increasing dyspnea and fatigue. **a** Contrast-enhanced axial CT image in mediastinal window setting depicts a massive pericardial effusion without prominent pericardial thickening, nodularities or enhancement. **b**, **c** Axial CT images at the level of the upper abdomen show an enlargement of the inferior vena cava with contrast reflux (*double yellow arrow*), periportal oedema (*yellow arrow*) and gallbladder oedema (*yellow asterisk*) as signs of right heart failure. Findings are compatible with cardiac tamponade. The woman was referred for urgent cardiac work-up and drainage of the cardiac fluid

Because of its high sensitivity for the detection of pericardial fluid, echocardiography is considered to be the primary imaging modality of choice to assess cardiac tamponade. Chest radiograph may show an enlarged cardiac silhouette with characteristic “water bottle” appearance. CT and magnetic resonance imaging (MRI) allow for a functional evaluation of the heart as well as characterisation of the pericardial effusion. CT findings of a thickened, enhancing or nodular pericardium and high-density fluid suggest a malignant pericardial effusion [20]. Signs suggestive of right heart failure are hepatic congestion and contrast reflux in the inferior vena cava and hepatic veins (Fig. 4). Other secondary findings indicative of a possible tamponade include enlargement of the SVC (diameter greater than the aorta), enlargement of the inferior vena cava (diameter greater than twice the adjacent aorta), periportal lymphoedema, angulation or bowing of the interventricular septum and flattening of the anterior surface of the heart (“flattened heart” sign) [21]. Though MRI is generally not used in the diagnosis of cardiac tamponade due to the emergent, life-threatening nature of the condition, MRI can be very useful in the evaluation of pericardial effusions, providing both morphological and functional information. MRI findings of a large haemorrhagic pericardial effusion with contrast-enhancing irregular or nodular pericardial thickening is suggestive of a metastatic pericardial effusion [22].

Since cardiac tamponade carries a high mortality, emergent pericardiocentesis, with or without placement of an indwelling pericardial drain, can be life-saving [17].

### Massive haemoptysis

Massive haemoptysis is defined as expectoration of 100 ml of blood in a single episode or more than 600 ml of blood over a 24-h period. Massive haemoptysis is a life-threatening medical emergency, which is fatal in about one-third of cases. Bronchogenic carcinoma is the most common cause of massive haemoptysis in patients over 40 years old with an overall rate of haemoptysis of 10-20%, although only fatal in 3% [23]. In case of severe haemoptysis, the bleeding usually stems from bronchial (90%) and pulmonary (5%) arteries [24, 25].

**Fig. 5:**
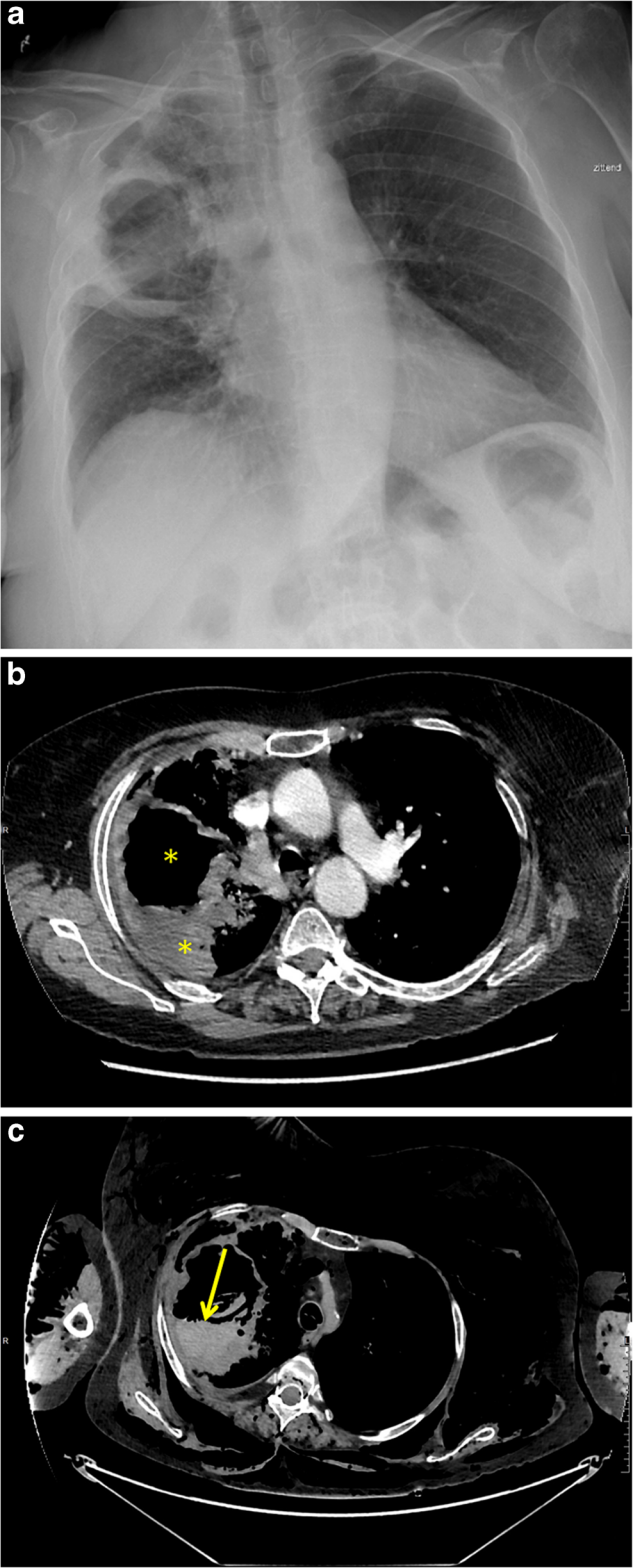
Massive haemoptysis and fatal air embolism in a 68-year-old woman with a cavernous lung lesion. **a** Anteroposterior chest radiograph, performed for inflammatory changes on blood tests and tachypnea, shows a giant cavitated consolidation in the right upper lobe. **b** Axial contrast-enhanced chest CT image confirmed a large ill-defined mass with central cavitation (*yellow asterisks*). Immediately following the CT, the patient developed massive haemoptysis in the radiology department necessitating intubation and ventilation. **c** Axial-contrast enhanced CT image of the chest after haemodynamic stabilisation shows a large amount of high-density fluid, compatible with blood (*yellow arrow*), in the cavern and the tracheobronchial tree. In addition, there was massive subcutaneous emphysema, a large amount of air in the vascular system and heart. Upon completion of the scan a fatal cardiac arrest ensued. Histopathology of the post-mortem examination confirmed a stage IV non-small cell lung cancer (poorly differentiated carcinoma)

Chest radiography might be the initial imaging modality since it is readily available. Chest radiography can assist in lateralising bleeding by demonstration of parenchymal and pleural abnormalities such as tumours and cavitary lesions. Multidetector CT may identify the bleeding site, cause and vascular origin of bleeding (bronchial arterial versus pulmonary arterial), while allowing a comprehensive evaluation of the lung parenchyma and mediastinum (Fig. 5). CT findings of active contrast extravasation, pseudo-aneursym formation and vessel invasion imply active bleeding [25]. Optimal arterial enhancement requires a tailored protocol with a region of interest positioned on the descending aorta. The scan should start during the peak enhancement (greater than 100 HU) after peripheral intravenous injection of a high-concentration contrast medium (350–400 mg/ml) at a flow rate of 3.5-5 mL/s [25].

In emergency, arterial endovascular embolisation is the procedure of choice while surgery may be performed in select cases when the patient is stabilised. Performing a multidetector CT angiography before endovascular treatment is useful to provide a detailed depiction of the origin and course of bronchial and non-bronchial systemic arteries responsible for haemoptysis and determine the optimal endovascular approach [25].

### Central airway obstruction

Central airway obstruction can be caused by a myriad of malignancies but is most commonly caused by lung cancer extending directly into the airway lumen. Up to 30% of lung cancer patients will have tumour obstruction of the central airways at some point in the course of their disease [26]. Central airway obstruction usually manifests with symptoms of respiratory distress, including stridor and dyspnoea, haemoptysis, cough and fever due to post-obstructive pneumonitis [20].

**Fig. 6:**
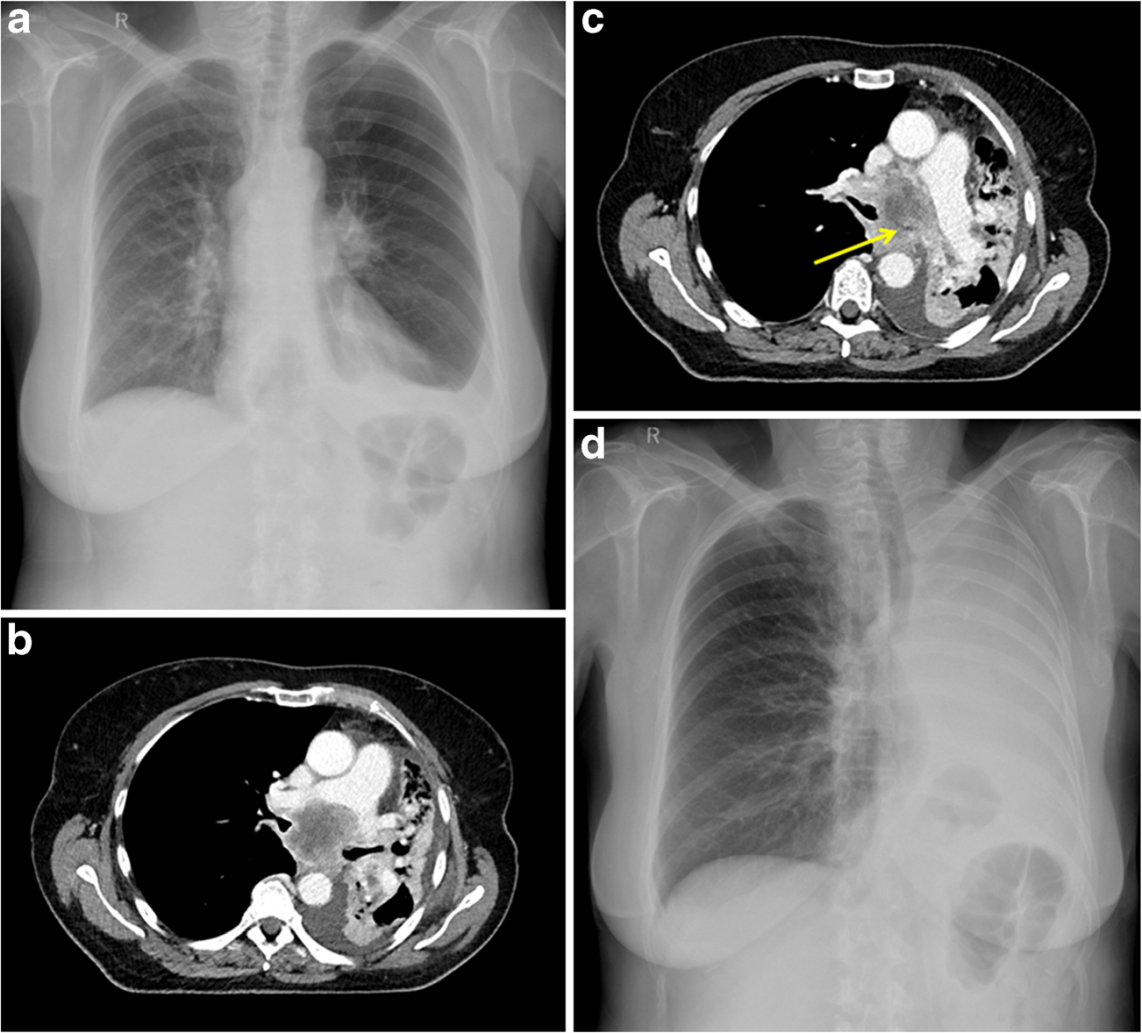
A 64-year-old woman was referred to the pulmonologist for persistent cough and increasing dyspnea. **a** Erect postero-anterior chest radiograph at the time of presentation shows an enlargement of the left hilum, air trapping in the left hemithorax (suggestive of partial bronchial obstruction) and a left-sided pleural effusion. **b** Axial contrast-enhanced chest CT in mediastinal window 1 day after the chest radiograph shows a left hilar mass with infracarinal extension, **c** partially obstructing the left main bronchus (*yellow arrow*). Although there is only a short time frame between the radiograph and the CT, there is marked volume loss of the left lung and mediastinal shift caused by retro-obstructive atelectasis. Diagnosis of stage IV non-small cell lung cancer was made. Four days after the CT examination, the patient experienced an acute episode of severe dyspnea and stridor for which she was referred to the emergency department. **d** Erect postero-anterior chest radiograph at admission shows a white lung on the left with massive mediastinal and cardiac shift, caused by complete obstruction of the left main bronchus. No air can be delineated in the left main bronchus. This was confirmed by bronchoscopy

Chest radiographs are non-sensitive and non-specific but may demonstrate tracheal narrowing or a hilar mass with retro-obstructive atelectasis or consolidation. The lateral radiograph can be of additional value because it provides a less obscured view of the trachea compared to the frontal view. Contrast-enhanced CT with coronal reconstructions is more sensitive and may identify the cause, site and severity of central airway obstruction and assess tumour extension (Fig. 6) [27]. Virtual bronchoscopy can be helpful for non-invasive evaluation of the tracheobronchial tree, allowing evaluation of the airways beyond a high-grade luminal obstruction not passable by a bronchoscope [28].

In case of severe airway obstruction, urgent therapeutic bronchoscopy with placement of airway stents is the treatment of choice [27]. To help determine the appropriate size of the stent, it is important to report the length of obstruction, the maximum degree of obstruction and the luminal diameter of the normal airways [29].

### Oesophagorespiratory fistula

An oesophagorespiratory fistula (ERF) is a rare, life-threatening complication of lung cancer affecting less than 1% of patients [30, 31]. The trachea is most commonly involved but oesophagobronchial and oesophagopulmonary fistula can also develop occasionally. An ERF, in the setting of lung cancer, may develop either through direct erosion of tumour through adjacent structures into the oesophagus or, uncommonly, after initial treatment, in particular in patients treated with angiogenesis inhibitors and chemoradiation [32]. ERF typically presents with coughing, dyspnoea secondary to aspiration pneumonitis, recurrent pulmonary infections and poor nutrition.

**Fig. 7:**
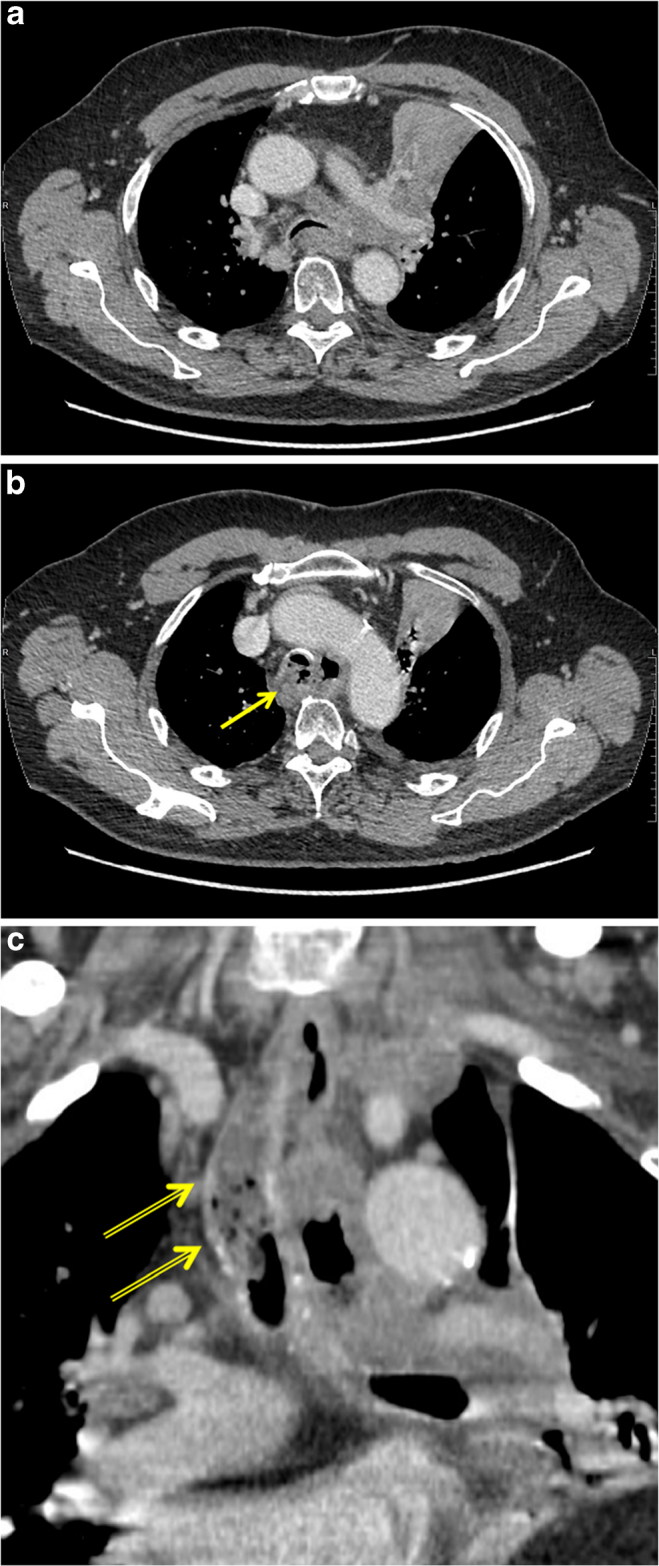
Tracheo-oesophageal fistula in a 60-year-old man with known stage IV non-small cell lung cancer who presented with a fever and had complaints of dysphagia. **a** Axial contrast-enhanced CT in mediastinal window setting shows a large left hilar mass encasing the left pulmonary artery with associated retro-obstructive atelectasis of the left upper lobe. Note mediastinal invasion extending to the trachea and oesophagus (*yellow arrow*). **b** Axial CT image at the level of the aortic arch reveals an extraluminal gas collection adjacent to both oesophagus and trachea. Both the walls of the oesophagus and trachea are thickened at this level with blurring and fatty infiltration of the surrounding fat planes. **c** Reformatted image in the coronal plane nicely depicts the presence of fluid, debris and air-bubbles in the trachea (*double yellow arrows*). The combination of findings is indicative of a tracheo-oesophageal fistula

Diagnosis is generally made clinically but can be confirmed on imaging. The imaging modality of choice is contrast oesophagography with a non-ionic water-soluble iodinated contrast medium. On CT, a direct communication between the oesophageal and airway lumens may be visualised, usually surrounded by soft-tissue thickening caused by underlying tumour (Fig. 7). In addition, orally ingested contrast material may be seen in the airway lumen and lung parenchyma. Multiplanar imaging with coronal and sagittal reconstructions may give more insight into the location and extent of the ERF. Associated findings, such as pulmonary consolidation, pleural fluid and abscess formation, can also be appreciated on CT.

Palliative stenting of the oesophagus and/or trachea is the treatment of choice [20].

## Neurological emergencies

### Malignant spinal cord compression

Malignant spinal cord compression (MSCC) is a common complication and has a negative effect on quality of life and survival. Because of rapid progression of neurological dysfunction, it is considered a medical emergency [33]. Between 2.5 and 5% of patients with terminal cancer will have MSCC, with lung cancer accounting for 15–20% of cases [34].

**Fig. 8:**
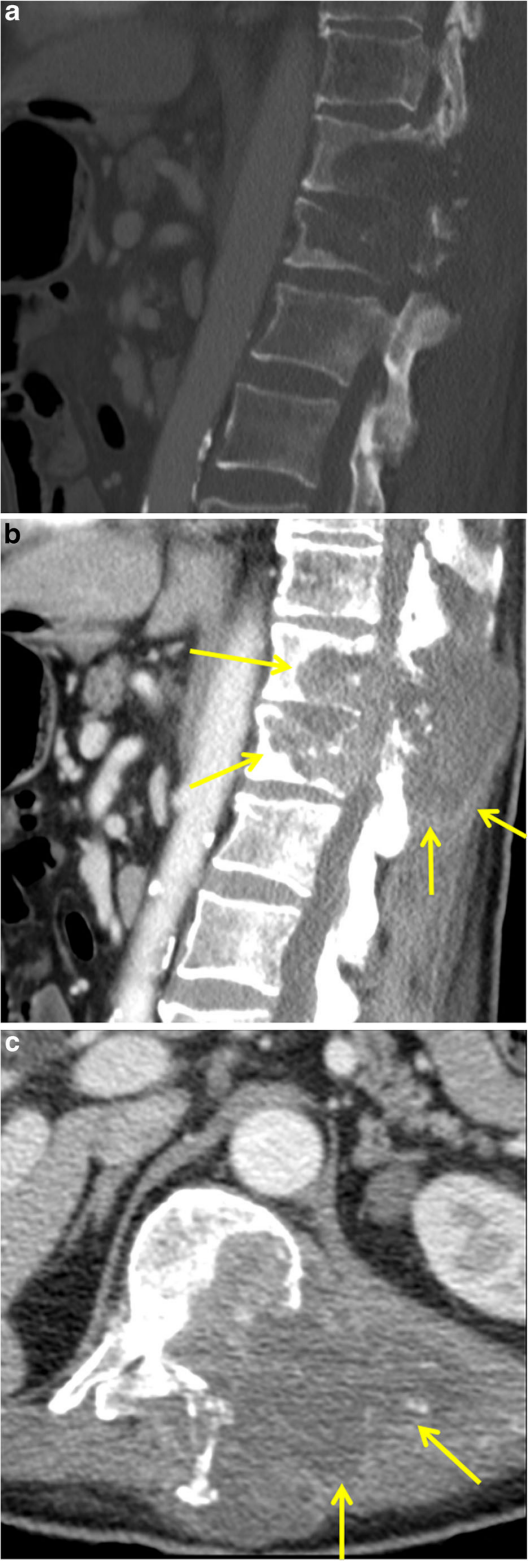
Malignant spinal cord compression of the thoracic spine in a 56-year-old man with stage IV non-small cell lung cancer. Sagittal enhanced CT image in bone (**a**) and soft-tissue (**b**) window setting at the thoracolumbar level shows confluent lytic bone lesions in the T11 and T12 vertebrae with disruption of the posterior wall and extending into the posterior elements. Also, note the large soft-tissue mass (*yellow arrows*) extending in the spinal canal resulting in a compression of the thoracic spinal cord. Axial enhanced CT image in soft tissue window (**c**) clearly shows the extension of the soft tissue mass in the spinal canal, while the spinal cord is no longer discernible

The most common cause of MSCC, accounting for over 85% of cases, is haematogenous spread of cancer cells to a vertebral body and epidural space. Subsequently, vertebral body collapse can cause spinal cord compression through posterior displacement with or without an associated epidural soft tissue mass. Other mechanisms of MSCC are direct tumour extension from a paraspinal mass or deposition of tumour cells in the spinal cord [34]. The thoracic spine is the most frequent site of MSCC, accounting for 70% of cases, followed by the lumbosacral (20%) and cervical spine (10%) [33]. Patients clinically present with back pain, by motor weakness (usually affecting the lower limbs), autonomic dysfunction and sensory loss [2]. Plain radiography is of limited use, since it can only detect vertebral body collapse, which is present in only 75% of patients with MSCC [33]. Because of the high soft-tissue resolution and multiplanar capability, MRI—with and without intravenous gadolinium administration—is the imaging modality of choice. It is more sensitive than CT in defining the local tumour extent and relationship with the spinal cord and can better distinguish benign from malignant causes of vertebral body collapse. A purely imaging-based six-point spinal cord compression grading scale was developed by Bilsky et al. [35], using T2-weighted MRI images: a grade of 0 indicates bone-only disease; 1a, epidural impingement without deformation of the thecal sac; 1b, deformation of the thecal sac, without spinal abutment; 1c, deformation of the thecal sac with spinal abutment, but without spinal compression; grade 2, spinal cord compression, but with CSF visible around the cord; grade 3, spinal cord compression, no CSF visible around the cord [33]. Both CT and MRI may show collapse of vertebral bodies and a spinal or paraspinal mass with compression and displacement of the thecal sack and spinal cord (Fig. 8).

Treatment options are corticosteroids, surgery and radiotherapy in different combinations [33].

### Carcinomatous meningitis

Carcinomatous meningitis occurs in approximately 5% of lung cancer patients, usually late in the course of the disease [36, 37]. Carcinomatous meningitis carries a poor prognosis with a high morbidity and survival ranging from weeks if untreated and up to 8 months with timely treatment [36]. Haematogenous dissemination is the most common mechanism of leptomeningeal tumour spread, but direct tumour extension from bone and brain lesions, and perivascular or perineural spread may also occur [38]. Symptoms of carcinomatous meningitis are myriad and include symptoms of cranial neuropathy, symptoms indicative of cerebral hemisphere involvement and symptoms of spinal cord or nerve root involvement [38].

**Fig. 9:**
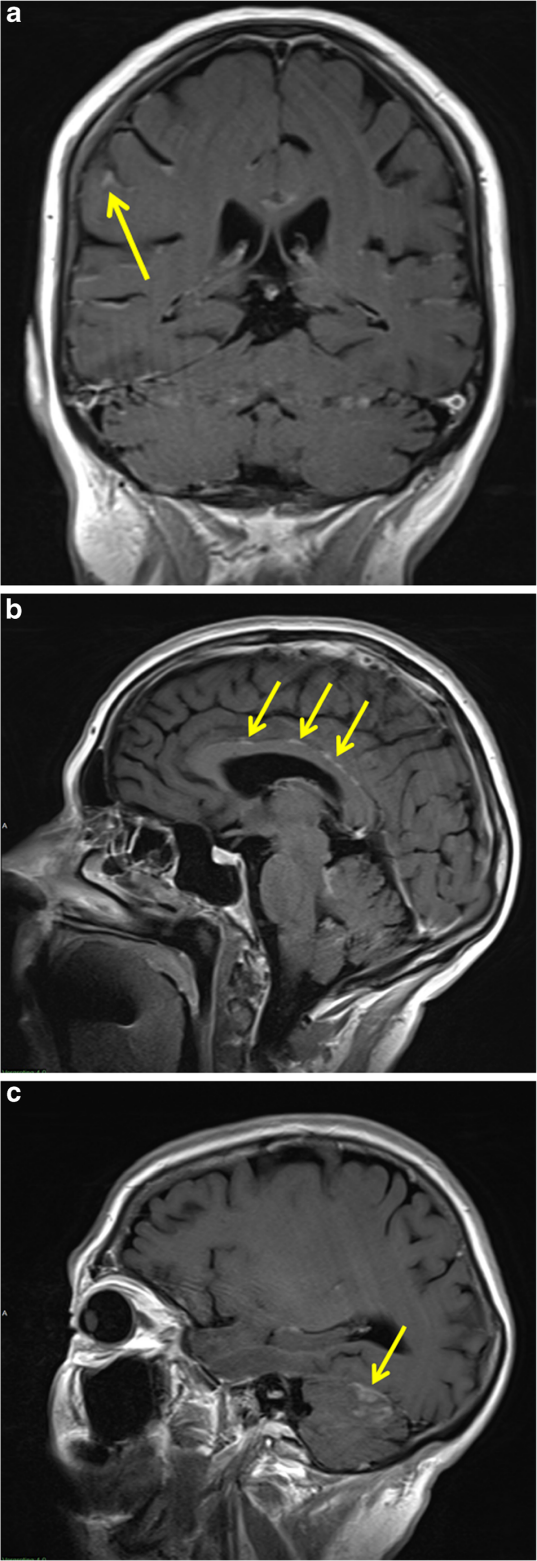
Carcinomatous meningitis in a 67-year-old woman with stage IV non-small cell lung cancer. Coronal (**a**) and sagittal (**b**, **c**) T1-weighted MR images after intravenous gadolinium contrast administration demonstrate diffuse, nodular leptomeningeal enhancement involving both cerebral hemispheres, but more markedly along the tentorium and into the subarachnoid spaces between the cerebellar folia (*yellow arrows*)

**Fig. 10:**
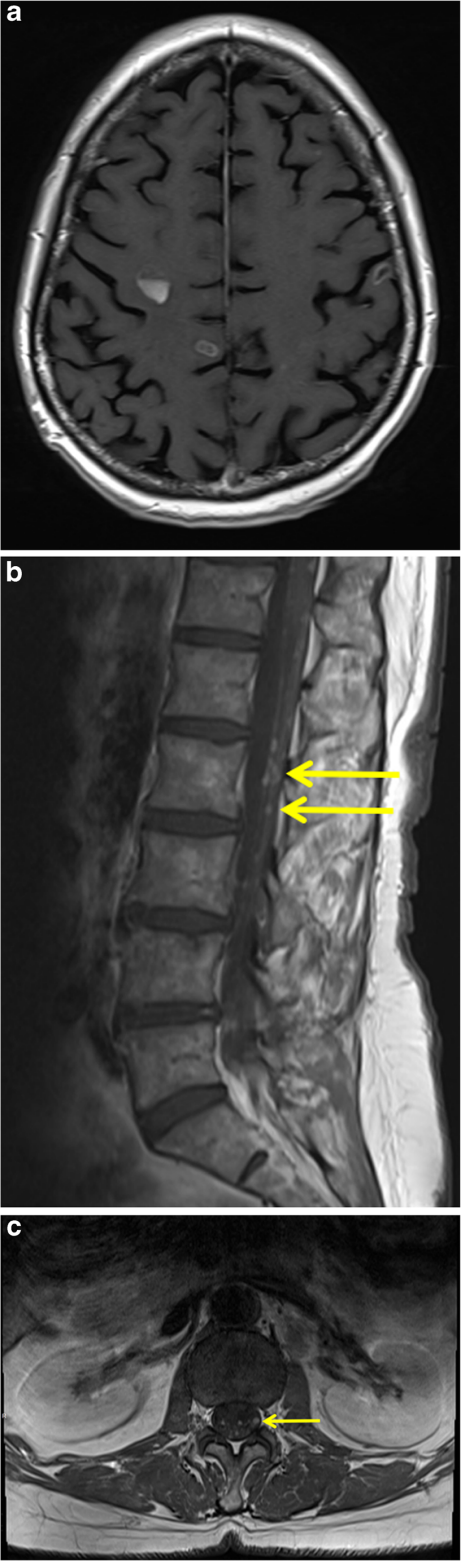
Intradural extramedullary metastases in a 54-year-old woman with non-small cell lung cancer. Axial T1-weighted image of the brain after intravenous gadolinium contrast administration (**a**) shows presence of multiple ring-enhancing parenchymal lesions consistent with cerebral metastases. Sagittal (**b**) and axial (**c**) T1-weighted MR images of the lumbar spine (after intravenous gadolinium contrast administration) demonstrate multiple enhancing tumour nodules (*yellow arrows*) along the lumbar spinal cord and cauda equina indicating leptomeningeal metastatic disease

Although cerebrospinal fluid (CSF) cytology remains the “gold standard” in confirming the diagnosis of carcinomatous meningitis, contrast-enhanced MRI is recommended to localise the disease sites before lumbar puncture or to support the diagnosis in suspected cases with negative cytology findings [37]. MRI may also demonstrate findings that may be a contra-indication for lumbar puncture, as well as associated abnormalities such as parenchymal metastases. Typical findings on MRI of carcinomatous meningitis are ependymal, leptomeningeal and dural enhancement (Fig. 9). Ependymal enhancement is characterised by a marked linear enhancement along the ventricular walls. Dural enhancement occurs adjacent to the inner table of the skull and does not extend into the sulci or cisterns. Leptomeningeal enhancement follows the pial surface of the brain and fills the subarachnoid spaces of the sulci and cisterns. This enhancement pattern is often described as having a “gyriform” or “serpentine” appearance. Furthermore, enhancement of cranial nerves, small superficial metastases in the sulci and ventricular dilatation in the setting of communicating hydrocephalus may be observed. Specifically, in the spinal canal, nodular enhancement, often in the cauda equina, is a key finding suggesting carcinomatous meningitis (Fig. 10) [36, 39]. Treatment should be guided by a multidisciplinary approach and includes surgery (ventriculo-peritoneal shunt or Ommaya reservoir placement), radiation therapy to high burden sites and both intrathecal and systemic chemotherapy [37].

### Cerebral herniation

**Fig. 11:**
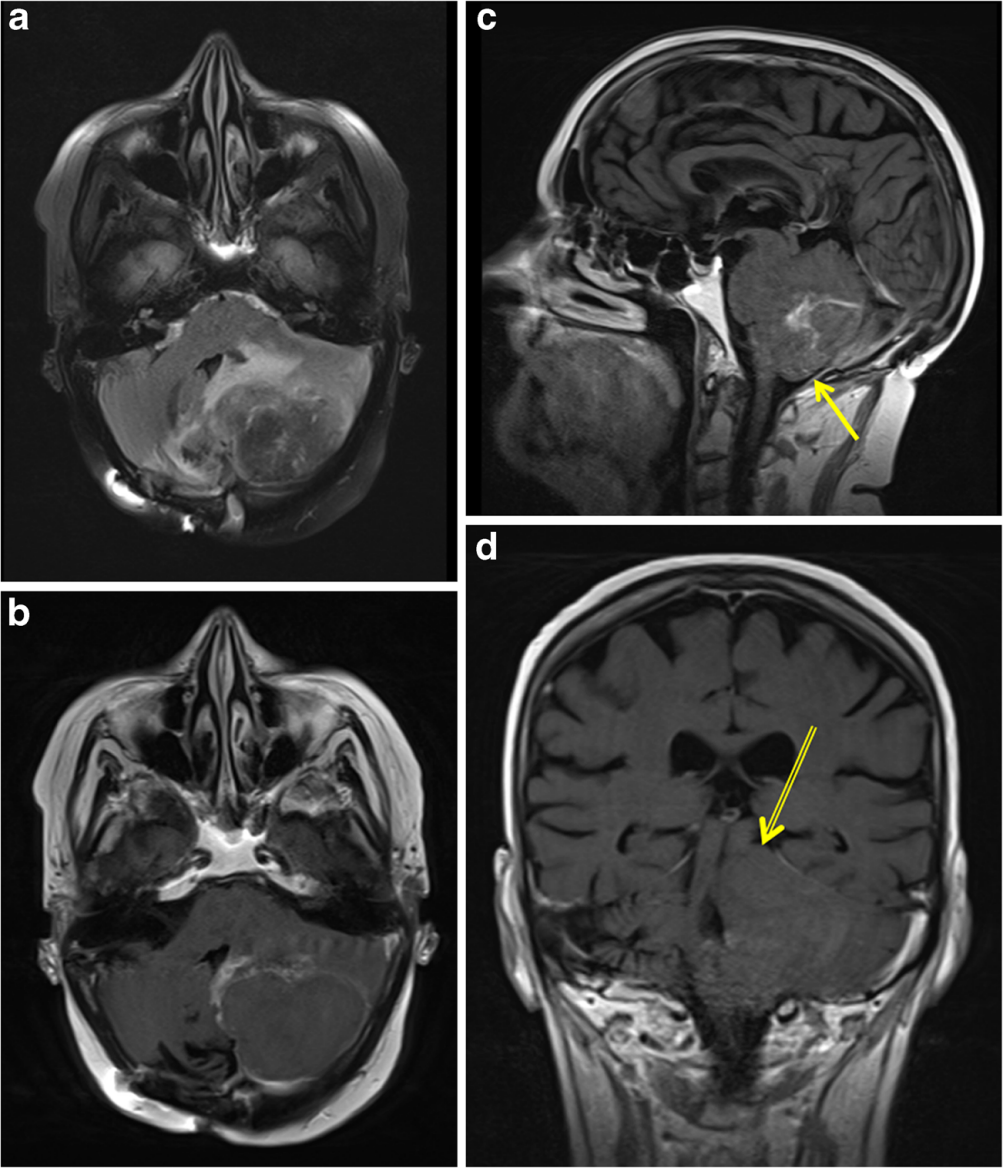
Cerebral herniation secondary to a cerebellar brain metastasis in a 51-year-old woman non-small lung cancer patient with a history of pancranial radiation therapy for brain metastases. Axial FLAIR (**a**) and axial T1-weighted image after intravenous gadolinium contrast administration (**b**) show a peripheral enhancing mass in the left cerebellar hemisphere with peritumoural oedema resulting in a midline shift and compression of the fourth ventricle. Sagittal T1-weighted contrast-enhanced image (**c**) demonstrates herniation of the left cerebellar tonsil trough the foramen magnum (*yellow arrow*). Coronal T1-weighted contrast-enhanced images shows an ascending transtentorial herniation with displacement of cerebellar tissue through the tentorial notch (*double yellow arrow*)

Intracranial metastasis, with lung cancer being the most common tumour of origin (20%), is the most frequent cause of cerebral herniation, an oncological emergency that should be rapidly assessed and promptly treated to avoid serious neurological sequelae or death [2, 40, 41]. Brain herniation occurs when an intracranial pathological process produces pressure that moves brain tissue from its normal anatomical location through rigid structures of the skull. Four distinct types of brain herniation have been associated with increased intracranial pressure: subfalcine, transalar (descending and ascending), transtentorial (ascending and descending) and tonsillar [41]. With subfalcine herniation, the most common type, the ipsilateral cingulate gyrus is displaced down and under the rigid midline falx with the risk of extensive cerebral infarction as a result of anterior cerebral artery compression. In descending transalar herniation, as a result of a frontal lobe mass effect, the posterior aspect of the orbital surface of the frontal lobe is displaced posteriorly and inferiorly over the sphenoid wing, risking to compress the middle cerebral artery. In ascending transalar herniation, the temporal lobe is displaced superiorly, potentially compressing the supraclinoid internal artery against the anterior clinoid process, resulting in an infarction of both the anterior and middle cerebral artery territories. Descending transtentorial herniation of the uncus across the tentorium cerebelli can cause compression of the oculomotor nerve (CN III). Ascending transtentorial herniation, caused by posterior fossa lesions, is most often symmetrical and can lead to obliteration of the perimesencephalic cisterns. Posterior cerebral artery vascular territory infarction and hydrocephalus as a result of Sylvian aqueduct compression can be observed in descending as well as ascending transtentorial herniation. Downward displacement of the cerebellar tonsils through the foramen magnum, most commonly caused by an infratentorial mass, may compress the medulla oblongata and compromise the respiratory centres as well as compress the posterior inferior cerebellar artery causing cerebellar infarcts [41]. CT is often the initial imaging modality in suspected central nervous system emergencies, since it is widely available and easily accessible. Nevertheless, contrast-enhanced MRI is the most sensitive and diagnostic tool available. Common findings on both CT and MRI in any type of brain herniation are displacement of brain parenchyma, midline shift and ventricular compression. Generally, these findings are best visualised on coronal MRI (Fig. 11) [41].

Treatment options include intravenous administration of mannitol or dexamethasone, which have a transient effect, or emergent neurosurgery as a more definite treatment modality [42].

## Musculoskeletal emergencies

### Pathological fracture

**Fig. 12:**
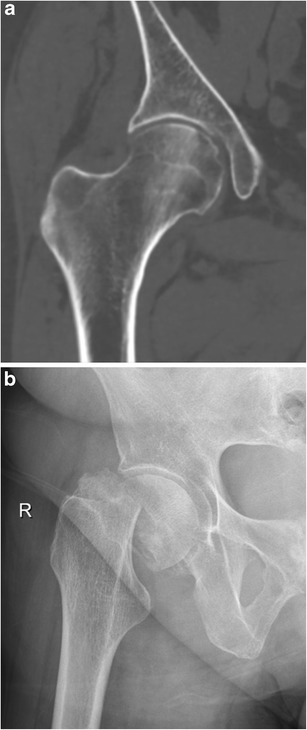
Subcapital insufficiency fracture of the right femur in a 77-year-old woman with stage IIIB non-small cell lung cancer. The patient developed extensive radiation pneumonitis, which was treated with high-dose glucocorticoids for 1 year, probably causing a greater rate of bone loss with subsequent insufficiency fracture. **a** Coronal non-enhanced CT image in bone window, performed 1 month prior to the fracture, shows no signs of metastatic bone involvement in the right hip. **b** Supine X-ray of the right hip shows a subcapital fracture of the right femur without associated osteolytic bone destruction. Absence of malignancy was confirmed on histopathological examination of the femoral head after total hip replacement

The bone is a very common site of metastatic disease, with lung cancer being the second most common primary tumour following breast cancer. Ultimately 30–65% of patients with metastatic lung cancer will have bone metastases and 9–29% of patients with bone metastases will develop a pathological fracture. Ninety percent of pathological fractures will need to be treated surgically [43]. Pathological fractures have a significant negative effect on quality of life and increase mortality [44]. A pathological fracture is defined as a fracture that occurs in abnormal bone, either malignant or non-malignant in nature. Non-malignant pathological fractures are mainly caused by osteoporosis induced by longstanding use of corticoid steroids (Fig. 12). Most common sites of a pathological fracture in metastatic tumours are the femur (44%), lumbar column (17%) and humerus (11%) [44]. A pathological fracture should be suspected when a fracture occurs after a minor trauma or during daily activities [45].

**Fig. 13:**
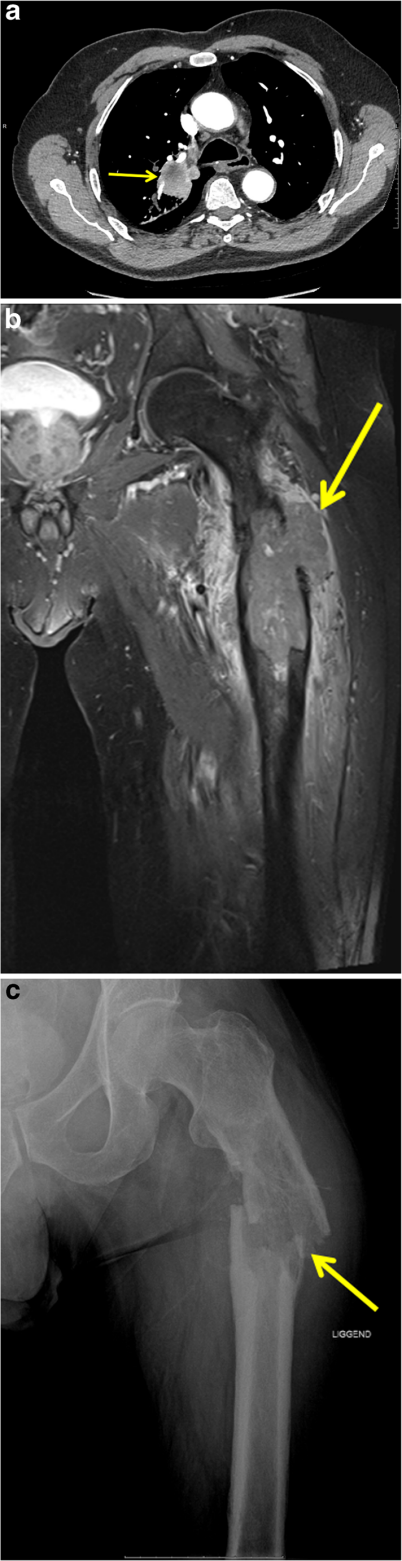
Pathological femoral shaft fracture in a 63-year-old man with a stage IV bronchogenic carcinoma. **a** Axial chest CT image in mediastinal window setting shows a large tumour in the right upper lobe (*yellow arrow*). **b** Coronal FS T1-weighted MR image after gadolinium contrast administration depicts a mass in the proximal left femoral shaft with extra-osseous soft tissue component (*yellow arrow*) and significant surrounding soft-tissue oedema. The patient presented to the emergency department 1 week after the MRI with complaints of extreme pain (non-traumatic) in the left hip and immobility. **c** Supine X-ray of the left femur reveals a pathological fracture with angulation at the level of the lytic bone metastasis (*yellow arrow*)

Conventional radiography is the first-line imaging modality of choice. Pathological fractures in lung cancer patients are typically caused by osteolytic metastasis, visible as radiolucent areas. CT can be useful in areas which are difficult to assess due to overlying bone structures such as the spine and pelvis. A mass around the fracture site (which is often better appreciated on MRI) is indicative of an underlying neoplasm (Fig. 13) [46]. In contrast to other primaries, lung cancer commonly spreads to the cortical bone with formation of the typical cortical metastases or so-called “cookie-bite lesions” [47].

Surgery is the treatment of choice, typically requiring plates or intramedullary rods with the addition of bone cement or joint arthroplasty when the fracture occurs near a joint [45].

## Conclusions

Due to often late-stage diagnosis with widespread metastatic disease and aggressive nature, oncological urgencies and emergencies are relatively frequent in the lung cancer patient. Whereas plain radiography often remains the first-line imaging modality that may point to possible abnormalities, multidetector CT with multiplanar imaging is the imaging modality of choice for urgencies and emergencies in the chest. In the central nervous system, this role is for MRI. Radiologists have a central and crucial role in the early recognition of these entities, as well as communication with thoracic oncologists, allowing appropriate management and minimising the risk of associated morbidity and mortality.

### Statement of authorship

This manuscript represents original work. Neither this manuscript nor one with substantially similar content has been published or is being considered for publication elsewhere. All authors contributed to this manuscript, read the manuscript and approved the final version of the submitted manuscript.

## Abbreviations

SVCS: Superior vena cava syndrome
SCLC: Small cell lung cancer
SVC: Superior vena cava
VTE: Venous thromboembolism
PE: Pulmonary embolism
CTPA: Computed tomography pulmonary angiography
ERF: Oesophagorespiratory fistula
MSCC: Malignant spinal cord compression
